# Understanding Despair: The Role of Physical Pain

**DOI:** 10.1177/08901171231177849

**Published:** 2023-05-18

**Authors:** Lucía Macchia

**Affiliations:** 1 4895 City, University of London, London, UK

**Keywords:** physical pain, deaths of despair, opioids, stress, premature mortality

## Abstract

In the United States, mortality due to alcohol, opioid overdose, and suicide has increased dramatically in the last decades. These deaths of despair have been the focus of recent and fast-growing literature. Yet little is known about the factors that are involved in despair. This article moves this area of research forward by highlighting the role that physical pain plays in the deaths of despair. This piece critically analyses the link between physical pain, the psychological states that precede pain, and the premature mortality that follows physical pain as well as the bidirectional relationships among these aspects.

The United States is one of the wealthiest and most developed nations in the world. Yet one issue is attracting the attention of policymakers and social scientists: mortality due to alcohol, opioid overdose, and suicide is rising at an alarming rate.^1^ For instance, in 2020 more than 93,000 Americans died from drug overdose, representing a 30% increase compared to 2019.^2^ These so-called deaths of despair demand urgent researchers’ and policymakers’ attention.

Although this topic has been addressed in recent literature,^1^ the lack of validated scales and established indicators of despair represents a challenge to the scientific community. In light of this situation, prior research has used wellbeing metrics as proxies for despair, namely negative emotions (e.g., sadness and stress), positive emotions (e.g., happiness), and the cognitive evaluation of one’s life (e.g., life satisfaction).^3-5^ However, despair seems to differ from these mental states. Despair comes from a Latin expression that means “absence of hope.” Following this definition, measures that captured people’s loss of confidence in the American Dream, and in opportunities for a better future have been used to understand despair.^6^ Given that previous studies focused on a selected set of metrics, there is the pressing need to consider other critical factors of the deaths of despair.

## The Present Article

Physical pain refers to unpleasant or uncomfortable sensations in the body.^7^ The link between pain and despair has already been suggested. However, how physical pain is related to despair and how pain research can help to understand the deaths of despair have received little attention in the scientific literature. This article has two aims: 1) to analyse the role that physical pain plays in other key aspects of despair, such as psychological states that involve desperation and despair-related causes of death, and 2) to highlight the importance of pain research in our understanding of the deaths of despair and in health promotion more broadly.

## Previous Research on Proxies for Despair

Prior work has used different metrics to understand despair. For instance, in an attempt to shed light on the increasing despair that American citizens were experiencing, Goldman et al.^4^ used measures of psychological distress and wellbeing from the Midlife in the United States (MIDUS) study. The authors found that the increasing trends in drug-related deaths were mirrored by growing trends in negative emotions (e.g., sadness, hopelessness, and worthlessness) and declining trends in positive emotions (e.g., happiness and fulfilment) and life satisfaction. Similarly, Blanchflower and Oswald^3^ used a measure of extreme distress and found that the percentage of US citizens in extreme distress nearly doubled from 1993 to 2019 and that this increase was greater among white, midlife, low-education individuals.

One step closer to the actual meaning of despair, other work has explored the link between lack of hope or optimism and despair-related causes of death. In one of many examples, Graham and Pinto^8^ found that the lack of optimism among American citizens was strongly linked to the increase in premature mortality. Cherlin^6^ proposed a different account: lack of hope in upward social mobility and fear of downward mobility may be determinants of despair. In a cohort study that followed 1154 individuals from early age to adulthood, Copeland et al.^9^ found that hopelessness, helplessness, low self-worth, and feeling unloved were associated with suicidal thoughts, illicit drugs, and opioid misuse but not with alcohol use disorder.

## The Link Between Pain and Despair

Physical pain has been found to be associated with psychological states that may involve feelings of despair, such as financial insecurity, lack of control, anxiety due to loss of work, and other negative emotions. Experimental studies demonstrated the link between physical pain and psychological factors. Chou et al.^10^ conducted five studies that showed that economic insecurity could lead to higher frequency of physical pain, more consumption of over-the-counter painkillers, and less pain tolerance. The authors suggested that financial insecurity may produce feelings of lack of control possibly promoting fear, anxiety, worry and, thus, physical pain. In a review paper, Wiech and Tracey^11^ described research that documented that experimentally induced mood changes and clinical mood disorders could predict physical pain. These studies showed that contextual stressors, such as worry and anxiety, may lead to new pain and increase existing pain. The review also showed that these effects were supported by neural mechanisms.

The link between physical pain and social stressors has also been documented using large-scale data. Wilkinson et al.^12^ used a longitudinal sample of 1113 individuals from the National Survey of Midlife Development in the United States to explore the link between economic recession-related stressors and chronic pain interference. The authors found that negative recession experiences, such as economic hardship, could increase pain interference. In line with this work, Macchia and Oswald^13^ used a sample of 1.3 million people from 146 countries, and fixed effects methods, and found that physical pain was lower in an economic boom and greater in an economic recession. Specifically, when the unemployment rate was high, physical pain was also high, suggesting that financial distress in periods of high global unemployment may create physical pain.

At the same time, pain is a key underlying factor in premature mortality. People tend to treat pain with painkillers, illicit drugs and alcohol, and unbearable pain is involved in suicide. In a detailed review of the literature on risk factors for suicidality in chronic pain, Tang and Crane^14^ concluded that the risk of committing suicide was doubled in chronic pain patients in comparison to non-pain patients. Some psychological processes, such as helplessness and hopelessness about pain, and pain-specific problems, such as insomnia due to pain, were other relevant risk factors for suicide.

Physical pain is also central in the US opioid epidemic. For more than four decades, opioids have been widely used to treat pain representing a challenge to the public health system. In a review article, Garland et al.^15^ has shown that the misuse of opioids prescribed to treat chronic pain causes changes in the brain that increase sensitivity to pain, negative affect, and tolerance to the medication reinforcing the cycle started by chronic pain. The authors stated that opioids relieve both physical pain and the psychological distress associated with pain reinforcing the drug habit even when it is no longer necessary. Building upon these relationships, Glei et al.^5^ explored the link between drug misuse, physical pain, and psychological distress. They found that physical pain was more strongly linked to drug misuse than to poor mental health and wellbeing (60% vs 19%).

**Figure 1.**
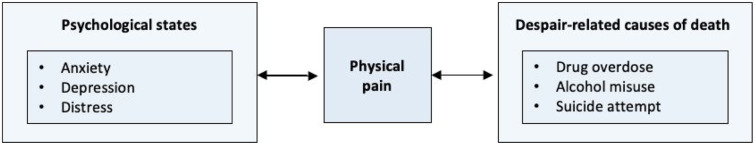
Relationship between psychological states, physical pain, and despair-related causes of death.

Feedback loops among physical pain, negative psychological states, and premature mortality have also been documented (See Figure 1). Negative psychological states can create physical pain which can increase depression and anxiety. Price^16^ conducted a detailed review of experimental and clinical studies that concluded that physical pain may lead to psychological disturbances, such as negative emotions and worry about future implications of the pain. This ‘secondary pain affect’ has been found to differ from pain unpleasantness and to be supported by different neural mechanisms.

Similarly, physical pain can lead to drug and alcohol misuse which tend to produce greater pain. Ballantyne and Mao^17^ conducted a detailed analysis of opioid therapy and concluded that the prolonged use of opioids painkillers tended to exacerbate physical pain instead of easing it.

These bidirectional relationships reinforce the link between psychological states, physical pain, and the causes of premature mortality creating a cycle with extremely negative consequences for people’s lives and overall wellbeing.

## Future Directions

Deaths of despair is one of the most challenging problems that researchers across the social and medical sciences are trying to understand. Measures of wellbeing, mental health, and optimism have been used as proxies for despair. Yet this research neglected one key aspect of despair: Physical pain. People’s pain levels have also been disregarded in other areas. For example, the massive prescription of opioids to treat pain has discouraged some physicians from prioritising people’s pain levels. However, scientific evidence suggests the opposite: The strong link of physical pain with psychological states and premature mortality highlights that people’s pain levels have valuable insights that can enrich our understanding of the deaths of despair.

These circumstances demand further research on pain. This work will not only uncover insights about the deaths of despair but also advance our knowledge about the causes and consequences of pain. Future research should also establish which type of pain can signal despair. It may be the case that occasional pain does not involve despair whereas chronic pain does. These aspects should be considered when examining the role of different types of pain in despair. Finally, given that physical pain is a great public health concern, pain research will also help to improve the design of health promotion and healthcare policies.

## References

[1] Case A Deaton A . Deaths of Despair and the Future of Capitalism. Princeton, NJ: Princeton University Press; 2020.

[2] Ahmad FB Rossen LM Sutton P . Provisional Drug Overdose Death Counts. Hyattsville, MA: National Center for Health Statistics; 2021. https://www.cdc.gov/nchs/nvss/vsrr/drug-overdose-data.htm

[3] Blanchflower DG Oswald AJ . Trends in extreme distress in the United States, 1993-2019. Am J Public Health. 2020;110(10):1538-1544. doi:10.2105/AJPH.2020.305811. 32816546 PMC7483105

[4] Goldman N Glei DA Weinstein M . Declining mental health among disadvantaged americans. Proc Natl Acad Sci U S A. 2018;115(28):7290-7295. doi:10.1073/pnas.1722023115. 29915079 PMC6048554

[5] Glei DA Stokes A Weinstein M . Changes in mental health, pain, and drug misuse since the mid-1990s: Is there a link? Soc Sci Med. 2020;246:112789. doi:10.1016/j.socscimed.2020.112789. 31978637 PMC7064160

[6] Cherlin AJ . Why are white death rates rising? The New York Times. 2016. https://www.nytimes.com/2016/02/22/opinion/why-are-white-death-rates-rising.html%0AIT’S

[7] Raja S Carr D Cohen M Finnerup NB Flor H Gibson S , et al. The revised International Association for the Study of Pain definition of pain: concepts, challenges, and compromises. Pain 2020;161(9):1976-1982. doi:10.1097/j.pain.0000000000001939. 32694387 PMC7680716

[8] Graham C Pinto S . Unequal hopes and lives in the USA: optimism, race, place, and premature mortality. J Popul Econ. 2019;32(2):665-733. doi:10.1007/s00148-018-0687-y.

[9] Copeland WE Gaydosh L Hill SN Godwin J Harris KM Costello J , et al. Associations of despair with suicidality and substance misuse among young adults. JAMA Netw Open 2020;3(6):e208627. doi:10.1001/jamanetworkopen.2020.8627. 32573708 PMC7312388

[10] Chou EY Parmar BL Galinsky AD . Economic insecurity increases physical pain. Psychol Sci. 2016;27(4):443-454. doi:10.1177/0956797615625640. 26893293

[11] Wiech K Tracey I . The influence of negative emotions on pain: Behavioral effects and neural mechanisms. Neuroimage. 2009;47(3):987-994. doi:10.1016/j.neuroimage.2009.05.059. 19481610

[12] Wilkinson LR Schafer MH Wilkinson R . How painful is a recession? An assessment of two future-oriented buffering mechanisms. Soc Sci Med. 2020;255:112455. doi:10.1016/j.socscimed.2019.112455. 32416438

[13] Macchia L Oswald AJ . Physical pain, gender, and the state of the economy in 146 nations. Soc Sci Med. 2021;287:114332. doi:10.1016/j.socscimed.2021.114332. 34500321

[14] Tang NKY Crane C . Suicidality in chronic pain: A review of the prevalence, risk factors and psychological links. Psychol Med. 2006;36(5):575-586. doi:10.1017/S0033291705006859. 16420727

[15] Garland EL Froeliger B Zeidan F Partin K Howard MO . The downward spiral of chronic pain, prescription opioid misuse, and addiction: cognitive, affective, and neuropsychopharmacologic pathways. Neurosci Biobehav Rev. 2013;37(10):2597-2607. doi:10.1016/j.neubiorev.2013.08.006. 23988582 PMC3967721

[16] Price DD . Psychological and neural mechanisms of the affective dimension of pain. Science. 2000;288(5472):1769-1772. doi:10.1126/science.288.5472.1769. 10846154

[17] Ballantyne J Mao J . Opioid therapy for chronic pain. N Engl J Med. 2003;349:1943-1953. doi:10.7326/l15-5109-3. 14614170

